# Supplementary material for: Causal Relationship between Aging and Anorexia Nervosa: A White-Matter-Microstructure-Mediated Mendelian Randomization Analysis
Source: Biomedicines. 2024 Aug 16;12(8):1874. doi: 10.3390/biomedicines12081874 (PMC11351342; doi:10.3390/biomedicines12081874)
Supplement: Supplementary file 1 [file biomedicines-12-01874-s001.zip › Supplementary Figure/Figure S8/Leaveoneout plot of MR analysis for TL to ubm-b-1461.pdf]

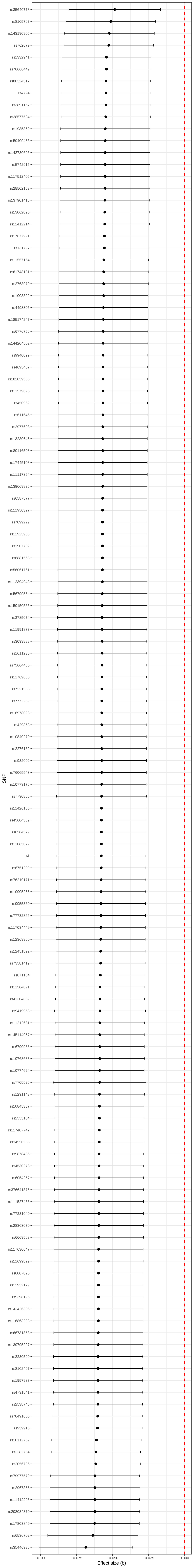
rs35640778
rs8105767
rs143190905
rs762679
rs1332941
rs76666449
rs80324517
rs4724
rs3891167
rs28577594
rs1985369
rs59409453
rs142730696
rs5742915
rs117512405
rs28502153
rs137901416
rs13062095
rs12412214
rs17677991
rs131797
rs11557154
rs61748181
rs2763979
rs1003322
rs4498805
rs185174247
rs6776756
rs144204502
rs9940099
rs4695407
rs182059586
rs11579626
rs450962
rs611646
rs2977608
rs13230646
rs80116508
rs17445108
rs11117354
rs139669835
rs6587577
rs111950327
rs7099229
rs12925933
rs1907702
rs6881568
rs56061761
rs112394943
rs56799554
rs150150565
rs3785074
rs11991877
rs3093888
rs1611236
rs75664430
rs11769630
rs7221585
rs7772289
rs16978028
rs429358
rs10840270
rs2276182
rs932002
rs76065543
rs10773176
rs7790856
rs11426156
rs45604339
rs6584579
rs11085072
All
rs6751209
rs76219171
rs10905255
rs9955360
rs77732866
rs117034449
rs12369950
rs12451892
rs73581419
rs871134
rs11584821
rs41304832
rs9419958
rs11212631
rs145114957
rs6790988
rs10768683
rs10774624
rs7705526
rs1291143
rs10845387
rs2555104
rs117407747
rs34550383
rs9878436
rs4530278
rs6054257
rs376641875
rs111527438
rs77231040
rs28363070
rs6669563
rs117630647
rs11699829
rs6007020
rs12932179
rs9398196
rs142426306
rs116863223
rs66731853
rs139795227
rs2230590
rs8102497
rs1957937
rs4731541
rs2538745
rs78491606
rs939916
rs10112752
rs2282764
rs2056726
rs79977579
rs2967355
rs11412296
rs202034370
rs17803849
rs6536702
rs35446936
SNP
-0.100
-0.075
-0.050
-0.025
0.000
Effect size (b)
